# Saunders’s Medical Hand-Atlases: Atlas and Epitome of Diseases of the Mouth, Pharynx and Nose

**Published:** 1903-07

**Authors:** 


Saunders's Medical Hand-Atlases:

Atlas and Epitome of Diseases of the Mouth, Pharynx and Nose. By Dr. L. Grunwald, of Munich. From the second revised and enlarged German edition. Edited, with additions, by James E. Newcomb, M. D., Instructor in Laryngology, Cornell University Medical School; Attending Laryngologist to the Roosevelt Hospital, Out-Patient Department. With 102 illustrations on 42 colored lithographic plates, 41 text-cuts and 219 pages of text. Philadelphia and London: W. B. Saunders & Co. 1903. [Cloth, $3.00 net.]

The value of this volume of the medical hand-atlas series is best indicated by the fact that a second edition has been found necessary, the first having been exhausted almost as soon as issued. In the second edition the author and editor have revised and enlarged the book, and it now stands unequalled as one cf the most perfect and valuable of the smaller works on the diseases of the mouth, pharynx and nose. Dr. Newcomb has added to the volume notes which represent the distinctively American views of the topics discussed by the author and these notes have added materially to the value of the work as an American publication. It now gives the best there is at the command of the famous German author, as well as the best there is in American ideas concerning the specialty treated of.

Much time and space have been devoted to the consideration of pathologic conditions and some of the common and yet very important operations have been described in detail, with a view to making their understanding perfectly clear. There is an enlargement of the illustrative feature of this edition, and there has been added a series of complete case histories which make a complete and very valuable section of the hand-atlas series.

